# Salicylic Acid Sustains Flower Performance Under Salinity in *Calendula officinalis* L. Plants Associated with ABA Responses and Antioxidant Regulation

**DOI:** 10.3390/plants15172599

**Published:** 2026-08-26

**Authors:** Xavier Rojas-Ruilova, María Rita Guzman, Isabel Marques

**Affiliations:** 1 Facultad de la Salud Humana, Universidad Nacional de Loja, C. Manuel Monteros, Loja 110103, Ecuador; xavier.rojas@unl.edu.ec; 2 Estación de Biodiversidad La Ceiba, Chisec 16013, Guatemala; 3 Forest Research Centre (CEF), Associate Laboratory TERRA, School of Agriculture (ISA), University of Lisbon, Tapada da Ajuda, 1349-017 Lisboa, Portugal

**Keywords:** salicylic acid, abscisic acid, flowering, redox homeostasis, salinity stress, reproductive performance, *Calendula officinalis*

## Abstract

Floral development and reproductive performance are among the most stress-sensitive processes in plants. Importantly, salicylic acid (SA) has emerged as an important regulator of abiotic stress responses in vegetative organs, but its role in maintaining flowering under salinity remains poorly understood. Here, we investigated the effects of exogenous SA (0.25–2.00 mM) on physiological, biochemical, and hormonal responses of *Calendula officinalis* L. exposed to 100 mM NaCl. Salinity significantly reduced vegetative growth, flower production, flower diameter, relative water content, and photosynthetic pigments, while increasing lipid peroxidation, proline accumulation, antioxidant enzyme activities, and abscisic acid (ABA) levels. SA alleviated these effects but in a dose-dependent manner. Under salinity, 0.25–0.50 mM SA improved vegetative and floral performance, with plant height, leaf number, and flower number increasing by up to 30.5%, 59.6%, and 100%, respectively. These responses were accompanied by a modulation of chlorophyll, carotenoid and malondialdehyde contents, as well as enzyme activities. However, high SA concentrations did not confer additional benefits and occasionally induced mild phytotoxic effects. SA-treated plants also exhibited enhanced ABA accumulation under salinity, supporting the involvement of ABA-associated responses to salinity. Collectively, our findings indicate that salicylic acid mitigates the detrimental effects of salinity on flower production and quality through coordinated modulation of antioxidant responses and ABA-associated responses.

## 1. Introduction

Flowers are among the most biologically and economically important structures in plants, serving as the foundation of sexual reproduction while determining the yield, quality, and market value of numerous horticultural crops. However, floral development and reproductive performance are highly sensitive to environmental stresses, often declining before severe damage becomes apparent in vegetative tissues [1]. Among abiotic stressors, salinity is particularly detrimental because it disrupts water relations, nutrient uptake and transport, and cellular homeostasis, ultimately impairing floral initiation, development, or quality. Salinity affects plants through a combination of osmotic stress, ion toxicity, and oxidative damage, leading to impaired growth, altered metabolism, and reduced reproductive success [2,3,4]. At the cellular level, excess salt promotes the accumulation of reactive oxygen species (ROS), which can damage lipids, proteins, and nucleic acids if not effectively controlled [5,6]. The cumulative effects of these physiological disturbances extend to plant reproductive development, one of the most sensitive phases of the plant life cycle [1]. Salinity can delay floral initiation and flower development, reduce flower production, impair pollen viability and anther dehiscence, and decrease stigma receptivity and pollen tube growth, ultimately compromising fertilization and reproductive success [7,8,9]. These detrimental effects are particularly important in ornamental species, where flower number, size, and longevity directly determine their commercial and aesthetic value [2]. For instance, salinity resulted in significant growth reduction and mortality in several *Zinnia* cultivars [10]. Even periwinkle (*Catharanthus roseus*) that exhibits some tolerance under moderate salinity (up to 3 dS m^−1^) shows a reduction in growth and flower production under salinity, as well as a decrease in the aesthetic value of flowers [11]. A recent comparative study of eight ornamental Asteraceae species also showed that, irrespective of species-specific differences, reproductive performance was consistently more sensitive to salinity than vegetative traits [12]. Despite these observations, little is known about the physiological, biochemical, and hormonal mechanisms associated with maintaining flowering under saline conditions. Identifying these responses is essential for understanding how plants can preserve reproductive performance when exposed to salinity.

Among the regulatory mechanisms involved in plant adaptation to stresses, phytohormones play a central role by coordinating growth, development, and stress responses. Acting as signalling molecules, they integrate environmental cues with physiological and metabolic adjustments, enabling plants to optimize resource allocation under adverse conditions [13]. Abscisic acid (ABA) has long been regarded as the central regulator of abiotic stress tolerance due to its role in stomatal closure, osmotic adjustment, and stress-responsive gene expression [14,15]. However, increasing evidence indicates that plant adaptation depends on the coordinated action of multiple hormones. In particular, salicylic acid (SA), jasmonic acid (JA), ethylene (ET), cytokinins, and auxins have emerged as crucial mediators of stress adaptation, operating in complex hormonal networks rather than in isolation [16,17].

Salicylic acid (SA) has attracted considerable attention because of its dual role in biotic and abiotic stress mitigation and its potential as a non-invasive and cost-effective management tool. Initially identified in the context of systemic acquired resistance (SAR) against pathogens and other biotic stressors [18], SA has since been implicated in tolerance to drought, salinity, extreme temperatures, and oxidative stress [19,20,21,22]. Under stress conditions, SA can improve plant performance by modulating antioxidant defenses, osmotic adjustment, and hormonal signaling. For example, SA promotes the activity of antioxidant enzymes such as superoxide dismutase (SOD), catalase (CAT), and ascorbate peroxidase (APX), thereby contributing to the maintenance of cellular redox balance [23,24,25]. In addition, SA influences osmolyte accumulation, including proline, which contributes to improved water-use efficiency and stress recovery by maintaining cell turgor and water uptake under conditions of low water potential [26,27]. In some species, SA modulates abscisic acid (ABA) signalling and guard cell turgor, triggering partial stomatal closure and reducing transpirational water loss, while still allowing some CO_2_ exchange for photosynthesis, helping maintain a positive carbon balance under water deficit. Consequently, exogenous SA application has shown beneficial effects in various species, including rice (*Oryza sativa*) [28], tomato (*Solanum lycopersicum*) [29], maize (*Zea mays*) [30], wheat (*Triticum aestivum*) [31] or grapes (*Vitis vinifera* × *Vitis labrusca*) [32] with improvements in growth, chlorophyll content, leaf relative water content (RWC), and yield components under stress. These responses are typically dose-dependent, with moderate concentrations (0.5–1.0 mM) eliciting beneficial effects, whereas excessive SA may become inhibitory or phytotoxic [33,34]. However, whether SA also contributes to the maintenance of flowering under saline conditions remains poorly understood. Addressing this gap would not only advance our understanding of the processes associated with maintaining reproductive performance under salinity but would also help optimize the use of SA as a sustainable strategy in ornamental horticulture.

Pot marigold (*Calendula officinalis* L.) is an ornamental and medicinal species of considerable economic importance, widely cultivated in temperate and Mediterranean regions for its ornamental value and as a source of bioactive compounds used in the pharmaceutical and food industries [35]. However, its cultivation is increasingly constrained by soil salinity and water scarcity, particularly in Mediterranean environments where saline irrigation is becoming more common. Previous studies have demonstrated that salinity markedly reduces flower production and quality in *C. officinalis*, together with pronounced physiological and biochemical alterations [35,36,37]. Thus, we investigated whether exogenous SA could mitigate the detrimental effects of salinity in *C. officinalis*. Specifically, we assessed the dose-dependent effects of SA on flowering, plant water status, redox homeostasis, and ABA accumulation under saline conditions.

## 2. Material and Methods

### 2.1. Plant Material and Growth Conditions

Seeds of *Calendula officinalis* L. (cv. Geisha) were surface-sterilized in 1% sodium hypochlorite for 5 min, rinsed thoroughly with sterile distilled water, and sown in 2 L pots containing a standardized substrate (50% peat, 25% perlite, 25% vermiculite; *v*/*v*/*v*). Germination and early seedling growth occurred in a controlled nursery environment, maintained at 20.5–25.3 °C, 72–76% relative humidity (RH), and a 16 h light/8 h dark photoperiod.

After two weeks, when seedlings had developed two true leaves, they were transplanted into individual 25 L pots filled with the same substrate and transferred to a controlled-environment growth chamber. Chamber conditions were maintained at 23 °C (day) and 19 °C (night), with 72–76% RH and a long-day photoperiod (16 h light). Photosynthetically active radiation (PAR) was kept between 700 and 800 μmol m^−2^ s^−1^ using a Tenney WITH-983 system (Tenney Environmental, New Columbia, PA, USA). Plants were irrigated twice weekly (50 mL per pot) with Hoagland nutritive solution containing: Na_2_Fe·EDTA (20 mg L^−1^), H_3_BO_3_ (2.86 mg L^−1^), MnSO_4_·4H_2_O (2.13 mg L^−1^), ZnSO_4_·7H_2_O (0.22 mg L^−1^), CuSO_4_·7H_2_O (0.08 mg L^−1^), and (NH_4_)_6_Mo_7_O_24_ (0.02 mg L^−1^). Before transplanting, substrate pH was adjusted to 5.7–5.9 using dilute KOH, and electrical conductivity (EC) standardized to 2.1 mS cm^−1^. Each pot contained a single plant and was placed on a polyethylene tray to ensure proper drainage. Plants were acclimated to these conditions for three weeks before starting salinity treatments.

### 2.2. Salinity-Stress Experiments

Experiments were conducted using one-month-old juvenile plants. Salinity was imposed through irrigation with sodium chloride (NaCl) at 100 mM NaCl, since a previous study showed marked physiological and growth impairments at this concentration [12]. A control group (0 mM NaCl) was irrigated only with sterile water. Saline solutions were prepared with sterile water, and each pot received 230 mL twice per week for 14 consecutive days. To avoid confounding effects of nutrient deficiency, all plants were additionally supplied with 80 mL of Hoagland nutritive solution at the end of each week. Electrical conductivity (EC) of the irrigation solutions was monitored throughout the experiment using a portable EC meter (Model HI98331, Hanna Instruments, Inc., Woonsocket, RI, USA), with the 100 mM NaCl treatment averaging 10.0 mS cm^−1^.

### 2.3. Salicylic Acid Application

SA was applied exogenously via foliar spray at five concentrations: 0.00 (control), 0.25, 0.50, 1.00, and 2.00 mM. These concentrations, selected from previous studies and arranged in a doubling series, were chosen to evaluate the dose-dependent effects of SA over a broad biologically relevant range, enabling the identification of both optimal and potentially inhibitory concentrations under saline conditions [38]. Following [38], stock solutions were prepared by dissolving SA in a minimal volume of ethanol and subsequently diluting by 1:1000 (*v*/*v* ethanol:water) with sterile distilled water to obtain the desired SA concentrations. To ensure consistent uptake, all plants were sprayed in the morning (08:00–09:00 h), avoiding peak transpiration periods. Applications were performed twice during the salinity stress period: (i) Day 0, at the onset of salinity treatment, to prime antioxidant and osmotic responses during early stress exposure, and (ii) Day 7, to reinforce potential protective mechanisms during mid-stress development. Plants were sprayed with approximately 100 mL of the appropriate SA solution per plant using a hand sprayer, ensuring uniform coverage of both the adaxial and abaxial leaf surfaces following the procedure of [39]. No SA was applied during the remaining experimental period to ensure that recorded effects reflected stress mitigation rather than recovery-driven repair. Possible phytotoxic effects were monitored by inspecting plants for symptoms such as leaf burn, necrosis, or growth inhibition. Each treatment included ten biological replicates, with all plants maintained under the same environmental conditions. Assessments were conducted on day 14. Fully expanded, healthy leaves from the upper third of the canopy were collected from each plant for all physiological, biochemical and hormonal analyses, frozen in liquid nitrogen, and stored at −80 °C until analyses.

### 2.4. Growth and Reproductive Performance

Morphological parameters were recorded to evaluate the cumulative impact of salinity stress on vegetative growth and reproductive performance. The following measurements were taken immediately prior to harvest: (i) Plant height, measured from the base of the stem (soil level) to the apex of the tallest inflorescence, expressed in centimeters (cm); (ii) Total number of leaves and flowers per plant, determined by counting all fully developed leaves and flowers present on each individual; (iii) Flower size, measured as the diameter (cm) of fully opened flowers, using three randomly selected flowers per plant to calculate the mean value. 

### 2.5. Biomass and Relative Water Content

At the end of the experimental period, the aboveground portion of each plant was harvested and immediately weighed to determine fresh weight (FW). The harvested material was then rehydrated in distilled water for 24 h at room temperature and weighed to determine turgid weight (TW). Subsequently, the same material was oven-dried at 70 °C for 48 h and weighed to determine dry weight (DW). Relative water content (RWC) was calculated to evaluate the plant’s water retention capacity under salinity stress, using the formula:

RWC (%) = [(FW – DW)/(TW – DW)] × 100

### 2.6. Photosynthetic Pigments

Leaf chlorophyll and carotenoid contents were determined following the method of [40] with slight modifications. Fresh leaf tissue (0.30 g) was used for pigment extraction, and a parallel subsample was oven-dried to constant weight to determine DW. For extractions, the fresh sample was weighed and homogenized in a pre-chilled mortar and pestle with 10 mL of 80% (*v*/*v*) acetone until a uniform slurry was obtained. The homogenate was transferred to 15 mL centrifuge tubes and centrifuged at 5000 rpm for 5 min at 4 °C to remove particulate debris. The supernatant was carefully collected, and the absorbance was measured using a UV–Vis spectrophotometer (Perkin-Elmer 2380, Springfield, IL, USA) at 663 nm, 645 nm, and 470 nm against 80% acetone as a blank.

Chlorophyll a (Chl a), chlorophyll b (Chl b), and carotenoid concentrations were calculated using the following equations:

Chlorophyll a (Chl a) = 12.7 × A_663_ – 2.69 × A_645_

Chlorophyll b (Chl b) = 22.9 × A_645_ – 4.68 × A_663_

Carotenoids = (1000 × A_470_ – 1.82 × Chl a – 85.02 × Chl b)/198

where A represents absorbance at the respective wavelength. All pigment contents were expressed as milligrams per gram of dry weight (mg g^−1^ DW).

### 2.7. Osmolyte Accumulation and Lipid Peroxidation

Proline content was determined using the acid-ninhydrin method, with toluene employed for chromophore extraction, as described by [41]. In brief, leaf tissue was homogenized in the extraction buffer and reacted with acid-ninhydrin reagent under heating to induce color development. After cooling, the mixture was extracted with toluene, and the absorbance of the resulting organic phase was measured spectrophotometrically at 520 nm. Proline concentrations were calculated using a standard curve and expressed as micrograms of proline per gram of dry weight (µg g^−1^ DW).

Lipid peroxidation was estimated by quantifying malondialdehyde (MDA) content using the thiobarbituric acid reactive substances (TBARS) assay, following the method of [42]. The absorbance of the pink-colored MDA–TBA complex was measured spectrophotometrically at 532 nm. MDA concentration was calculated using an extinction coefficient of 155 mM^−1^ cm^−1^ and expressed as nanomoles of MDA per gram of dry weight (nmol g^−1^ DW).

### 2.8. Antioxidant Enzyme Activities

To evaluate the antioxidant responses under salinity stress, leaf samples were homogenized in an extraction buffer containing 200 mM Tris-HCl (pH 8.0), 10 mM MgCl_2_·6H_2_O, 30 mM β-mercaptoethanol, 4 mM dithiothreitol (DTT), 2% (*v*/*v*) Triton X-100, 10% (*v*/*v*) glycerol, and a protease inhibitor cocktail (EDTA-free; Complete™, Roche, Basel, Switzerland; 2 tablets per 50 mL). To remove phenolic compounds, 1% (*w*/*v*) polyvinylpolypyrrolidone (PVPP) was added during homogenization. The homogenates were centrifuged at 13,000× *g* for 20 min at 4 °C, and the resulting supernatants were used for enzymatic assays.

Catalase (CAT) activity was determined in a 1.5 mL reaction mixture containing 10 mM H_2_O_2_ and 50 mM phosphate buffer (pH 7.0). The enzyme extract was added immediately before initiating the reaction, and the decomposition of H_2_O_2_ was monitored spectrophotometrically at 240 nm, as described by [43]. Enzyme activity was calculated from the rate of decrease in absorbance and expressed as CAT units per mg of protein dry weight (DW), where one unit corresponds to the decomposition of 1 mM H_2_O_2_ min^−1^.

Peroxidase (POD) activity was assayed by measuring the rate of H_2_O_2_-dependent oxidation of the substrate at 430 nm, following the method of [44]. Activity was calculated using an extinction coefficient of 2.47 mM^−1^ cm^−1^, with one POD unit defined as the amount of enzyme catalyzing the decomposition of 1 µmol H_2_O_2_ min^−1^. Results are expressed as units per mg of protein DW.

Ascorbate peroxidase (APX) activity was measured spectrophotometrically at 290 nm in a 1 mL reaction mixture containing 20 mM ascorbate, 0.1 mM H_2_O_2_, 50 mM phosphate buffer (pH 7.8), and 10 µL of enzyme extract, according to [45]. The decrease in absorbance due to ascorbate oxidation was quantified using an extinction coefficient of 2.8 mM^−1^ cm^−1^. Results are expressed as APX units per mg of protein DW.

### 2.9. Abscisic Acid (ABA) Content

Abscisic acid (ABA) content in leaf tissue was determined using high-performance liquid chromatography (HPLC). Fresh leaf samples (0.30 g) were flash-frozen in liquid nitrogen, ground to a fine powder, and homogenized in 5 mL of cold 80% (*v*/*v*) methanol containing 1 mM butylated hydroxytoluene (BHT) to prevent oxidative degradation. The homogenates were vortexed for 1 min and incubated at 4 °C for 12 h in darkness, followed by centrifugation at 10,000× *g* for 20 min at 4 °C. The supernatant was collected, and the pellet was re-extracted once under the same conditions. Combined supernatants were filtered through a 0.22 µm polytetrafluoroethylene (PTFE) syringe filter before injection into the HPLC system. HPLC analysis was performed on a Shimadzu LC-20AT system (Shimadzu, Kyoto, Japan) equipped with two pumps and a photodiode array (PDA) detector, using a reverse-phase C18 column (250 × 4.6 mm, 5 µm particle size; Waters, Milford, MA, USA). The mobile phase consisted of solvent A (0.1% formic acid in water) and solvent B (acetonitrile), with a linear gradient from 25% to 60% B over 15 min at a flow rate of 1.0 mL min^−1^. Detection was performed at 254 nm. ABA identification was confirmed by comparing retention times and UV absorption spectra with those of authentic ABA standards (Sigma-Aldrich, St. Louis, MO, USA). ABA was quantified using a calibration curve prepared with known concentrations of authentic ABA standard, and the ABA concentration in the leaf extracts was expressed as ng mL^−1^.

### 2.10. Statistical Analysis

All data are presented as mean values ± standard error (SE), calculated from ten biological replicates per treatment. Statistical analyses were conducted using IBM SPSS Statistics software, version 28. Prior to analysis, data were tested for normality using the Shapiro–Wilk test and for homogeneity of variances using Levene’s test. As the assumptions required for the analysis were adequately satisfied, no data transformation was necessary. To evaluate the effects of salinity level, SA concentration, and their interaction, a two-way analysis of variance (ANOVA) was performed for each measured variable, with salinity and SA included as fixed factors. When a significant interaction was detected, simple effects were examined using estimated marginal means from the factorial model. Pairwise comparisons among SA concentrations within each salinity level and between salinity levels within each SA concentration were adjusted for multiple comparisons using the Sidak procedure. Statistical significance was accepted at *p* < 0.05.

## 3. Results

### 3.1. Plant Growth and Morphological Parameters

**Figure 1:**
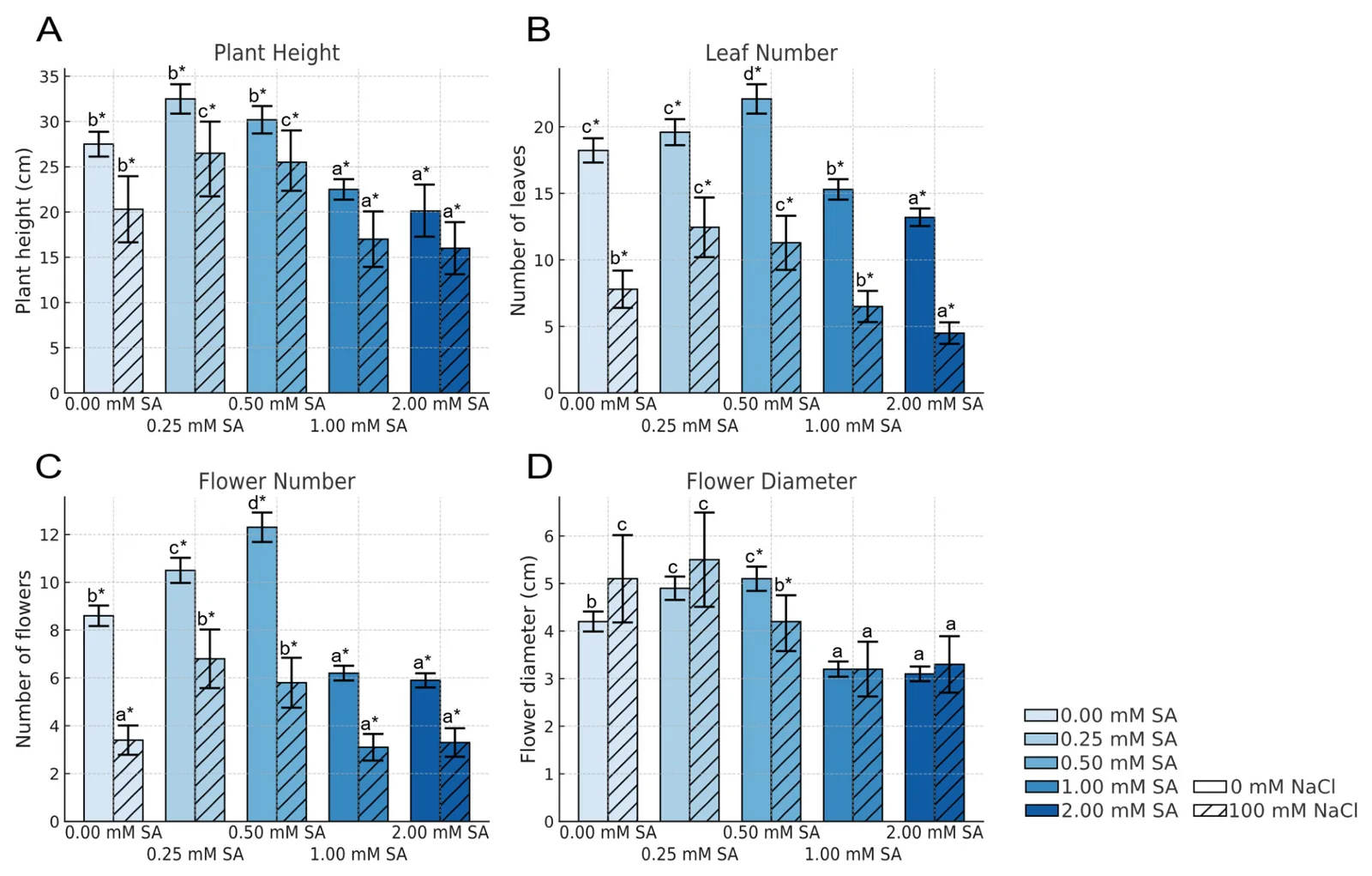
Morphological responses of *Calendula officinalis* L. to combined salinity (NaCl) and salicylic acid (SA) treatments. Bars represent mean ± SE (*n* = 10) for plant height (**A**), number of leaves (**B**), number of flowers (**C**), and flower diameter (**D**) grown under two NaCl levels (0 mM and 100 mM) and five SA concentrations (0.00, 0.25, 0.50, 1.00, or 2.00 mM). Blue shading intensity reflects increasing SA concentration (0.00–2.00 mM), and hatching distinguishes NaCl treatments (no hatching = 0 mM NaCl; hatching = 100 mM NaCl). Different lowercase letters indicate significant differences among SA concentrations within the same NaCl level, whereas asterisks indicate significant differences between NaCl levels within the same SA concentration (*p* < 0.05).

In the absence of salinity (0 mM NaCl), plants maintained high growth, although significant differences were reported among SA doses (Figure 1). The foliar application of 0.25 mM SA significantly increased plant height (F_4,90_ = 8.23, *p* < 0.001), the number of leaves (F_4,90_ = 6.27, *p* < 0.001) and flowers (F_4,90_ = 7.05, *p* < 0.001), as well as their size (F_4,90_ = 5.29, *p* < 0.001). The 0.50 mM SA treatment further enhanced all these traits. By contrast, higher SA concentrations (1.00 and 2.00 mM) reduced all traits (Figure 1).

Salinity also affected plant height (F_1,90_ = 15.61, *p* < 0.001), the number of leaves (F_1,90_ = 18.27, *p* < 0.001) and flowers (F_1,90_ = 22.78, *p* < 0.001) and their size (F_1,90_ = 13.04, *p* < 0.001). Under salinity (100 mM NaCl), plants without SA supplementation showed significant reductions compared with the non-saline (Figure 1). For instance, compared with plants exposed to 100 mM NaCl without SA, application of 0.25 mM SA increased plant height, leaf number, and flower number by approximately 30.5%, 59.6%, and 100.0%, respectively, while flower diameter was 7.8% higher. Application of 0.50 mM SA increased plant height, leaf number, and flower number by approximately 25.6%, 44.7%, and 70.6%, respectively. In contrast, the higher SA concentrations (1.00 and 2.00 mM) did not alleviate the salinity-induced reductions and, for several traits, further reduced plant performance. Significant NaCl × SA interactions were observed for plant height (F_4,90_ = 5.41, *p* < 0.001), leaf number (F_4,90_ = 6.23, *p* < 0.001), flower number (F_4,90_ = 5.99, *p* < 0.001), and flower diameter (F_4,90_ = 7.34, *p* < 0.001).

### 3.2. Leaf Water Status

**Figure 2:**
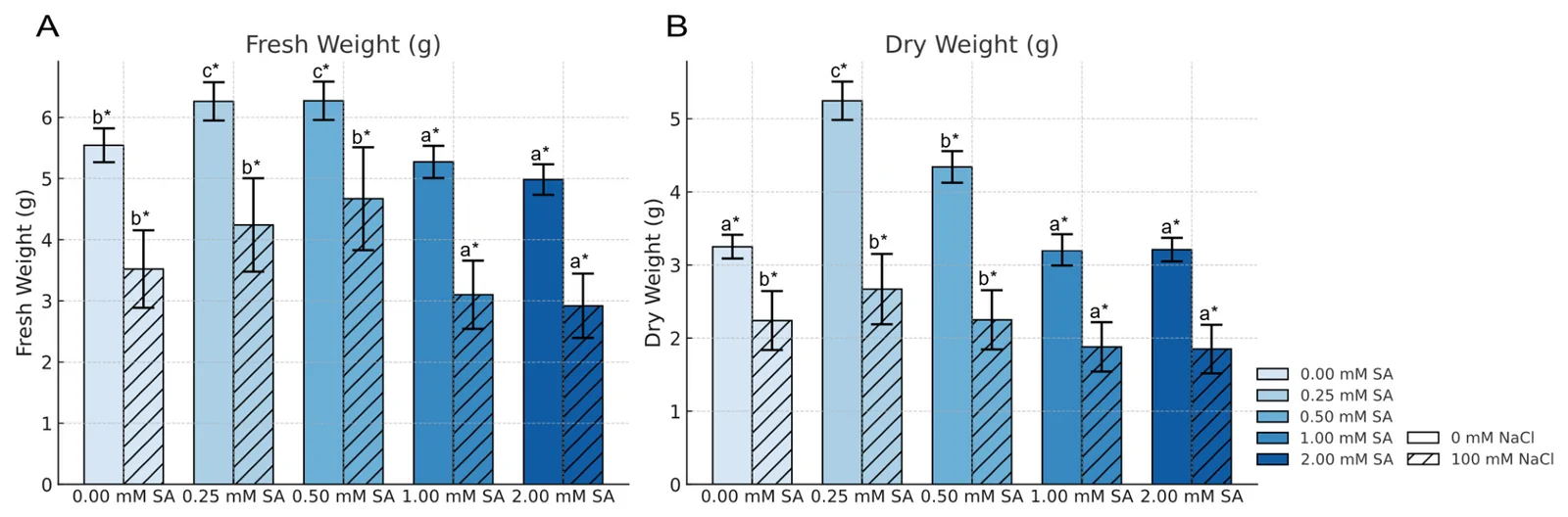
Plant biomass considering fresh weight (FW) (**A**) and dry weight (DW) (**B**) of *Calendula officinalis* plants grown under two NaCl levels (0 mM and 100 mM) and five SA concentrations (0.00, 0.25, 0.50, 1.00, or 2.00 mM). Bars represent mean ± SE (*n* = 10). Different lowercase letters indicate significant differences among SA concentrations within the same NaCl level, whereas asterisks indicate significant differences between NaCl levels within the same SA concentration (*p* < 0.05).

In the absence of salinity, SA significantly affected biomass in a concentration-dependent manner, either considering FW or DW values (respectively, F_4,90_ = 15.14, *p* < 0.001 and F_4,90_ = 11.18, *p* < 0.001). The application of 0.25 mM SA promoted the highest FW and DW values, whereas higher SA concentrations (1.00 and 2.00 mM) significantly reduced both traits (Figure 2). The levels of salinity also affected significantly FW (F_1,90_ = 5.78, *p* < 0.05) and DW (F_1,90_ = 6.29, *p* < 0.05) values. In the absence of SA, exposure to 100 mM NaCl reduced FW and DW by approximately 36% and 30%, respectively, compared with the corresponding non-saline treatment (Figure 2). Under salinity, 0.25 mM SA significantly increased both FW and DW relative to the saline treatment without SA, whereas 0.50 mM SA significantly increased FW but not DW. In contrast, 1.00 and 2.00 mM SA resulted in significantly lower biomass values under saline conditions (Figure 2). Significant NaCl × SA interactions were observed for FW (F_4,90_ = 13.56, *p* < 0.001), and DW (F_4,90_ = 11.27, *p* < 0.001).

Under non-saline conditions, RWC declined progressively with increasing SA concentration, ranging from 97.6% in untreated plants to 61.4% at 2.00 mM SA. Under salinity, RWC decreased markedly compared with non-saline conditions, reaching 67.9% in untreated plants. Foliar application of 0.25 mM SA resulted in only a slight increase in RWC (70.5%), whereas 0.50 mM SA substantially increased RWC, reaching 84.9%. In contrast, higher SA concentrations (1.00 and 2.00 mM) markedly reduced RWC to 32.8% and 35.9%, respectively.

### 3.3. Photosynthetic Pigments

**Figure 3:**
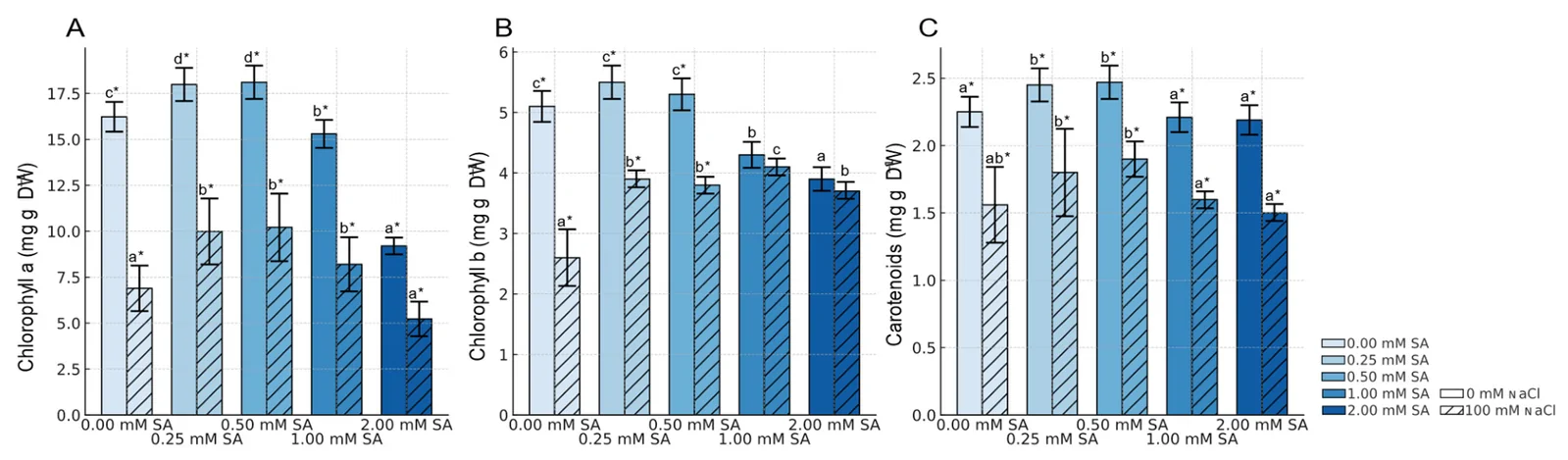
Levels of chlorophyll a (**A**), chlorophyll b (**B**), and carotenoid content (**C**) measured in *Calendula officinalis* leaves grown under two NaCl levels (0 mM and 100 mM) and five SA concentrations (0.00, 0.25, 0.50, 1.00, or 2.00 mM). Bars represent mean ± SE (*n* = 10). Different lowercase letters indicate significant differences among SA concentrations within the same NaCl level, whereas asterisks indicate significant differences between NaCl levels within the same SA concentration (*p* < 0.05).

Salinity had a significant effect on the levels of Chl a (F_1,90_ = 24.05, *p* < 0.001), Chl b (F_1,90_ = 26.07, *p* < 0.001), and carotenoid contents (F_1,90_ = 29.76, *p* < 0.001), decreasing all photosynthetic pigments measured (Figure 3). Under non-saline conditions, the response to SA was concentration-dependent, with the highest pigment contents generally observed at the lower SA concentrations (0.25–0.50 mM), whereas higher concentrations resulted in lower values (Figure 3). Under salinity, low SA concentrations partially mitigated pigment loss, although the response differed among pigments and SA concentrations. In contrast, 1.00 and 2.00 mM SA generally provided little or no improvement over the saline treatment without SA (Figure 3). A significant effect was therefore detected depending on SA concentrations (Chl a: F_4,90_ = 8.92, *p* < 0.001), Chl b (F_4,90_ = 8.03, *p* < 0.001), and carotenoid contents (F_4,90_ = 11.01, *p* < 0.001). These contrasting responses also accounted for the significant NaCl × SA interactions observed for Chl a (F_4,90_ = 15.19, *p* < 0.001), Chl b (F_4,90_ = 16.28, *p* < 0.001), and carotenoid contents (F_4,90_ = 19.26, *p* < 0.001).

### 3.4. Osmolyte Accumulation and Lipid Peroxidation

**Figure 4:**
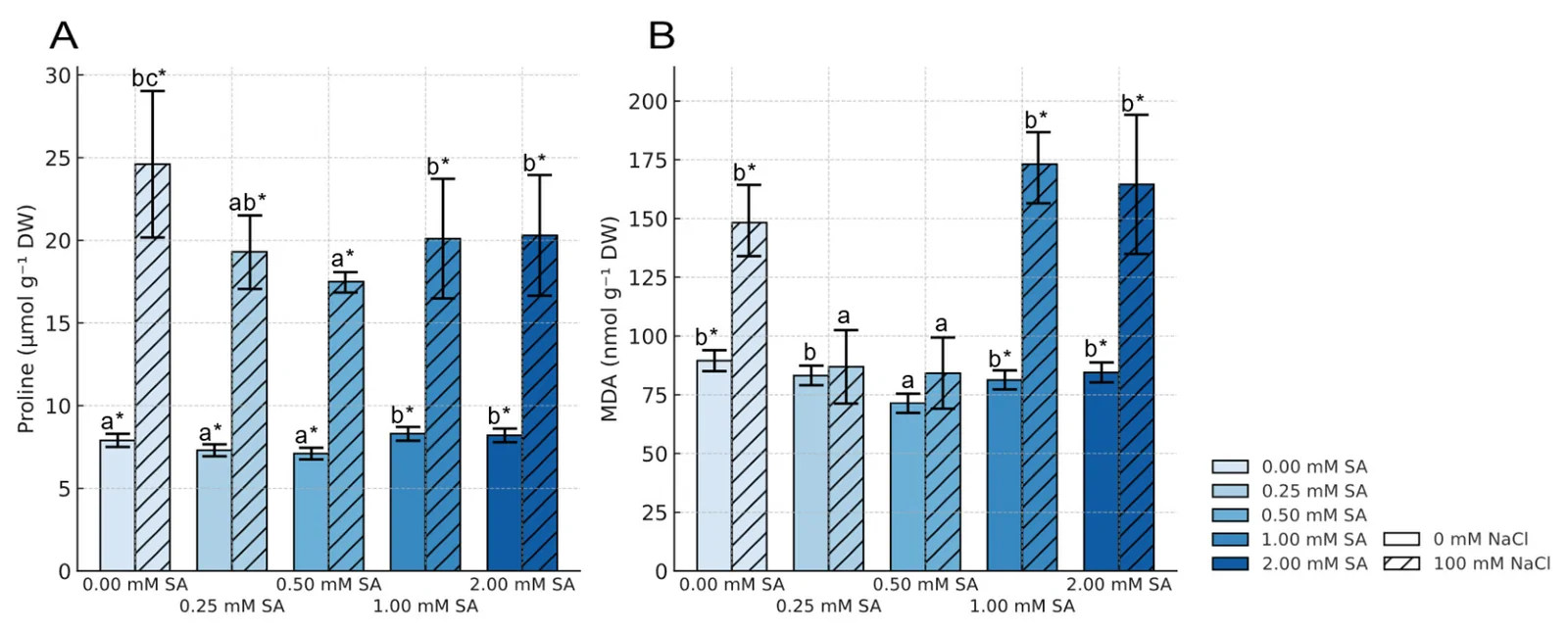
Proline (**A**) and malondialdehyde (MDA) (**B**) concentrations measured in *Calendula officinalis* leaves grown under two NaCl levels (0 mM and 100 mM) and five SA concentrations (0.00, 0.25, 0.50, 1.00, or 2.00 mM). Bars represent mean ± SE (*n* = 10). Different lowercase letters indicate significant differences among SA concentrations within the same NaCl level, whereas asterisks indicate significant differences between NaCl levels within the same SA concentration (*p* < 0.05).

Salinity markedly increased proline accumulation, with values remaining significantly higher than those of the corresponding non-saline treatments (F_1,90_ = 16.78, *p* < 0.001) (Figure 4). The application of SA had a significant effect on proline although its contents varied across concentrations (F_4,90_ = 5.21, *p* < 0.001). For instance, under non-saline conditions, proline content remained low and did not differ significantly among 0, 0.25, and 0.50 mM SA treatments, whereas significantly higher values were observed at 1.00 and 2.00 mM SA (Figure 4). In contrast, under salinity, the lowest proline levels were observed at the lower SA concentrations, particularly 0.25 and 0.50 mM, with proline accumulation increasing again at 1.00 and 2.00 mM SA (Figure 4).

MDA content was also strongly affected by salinity (F_1,90_ = 11.25, *p* < 0.001) and SA concentration (F_4,90_ = 22.17, *p* < 0.001). Under non-saline conditions, low SA concentrations resulted in lower MDA levels, whereas higher SA concentrations showed no beneficial effect (Figure 4). Salinity markedly increased MDA content in plants without SA supplementation. Under saline conditions, the application of 0.25 or 0.50 mM SA significantly reduced MDA accumulation relative to the saline treatment without SA, while higher SA concentrations (1.00 and 2.00 mM) were associated with higher MDA levels (Figure 4). The significant NaCl × SA interactions observed for proline (F_4,90_ = 19.23, *p* < 0.001), and MDA (F_4,90_ = 21.78, *p* < 0.001) further suggested the concentration-dependent nature of the SA response under salinity.

### 3.5. Antioxidant Enzyme Activity

**Figure 5:**
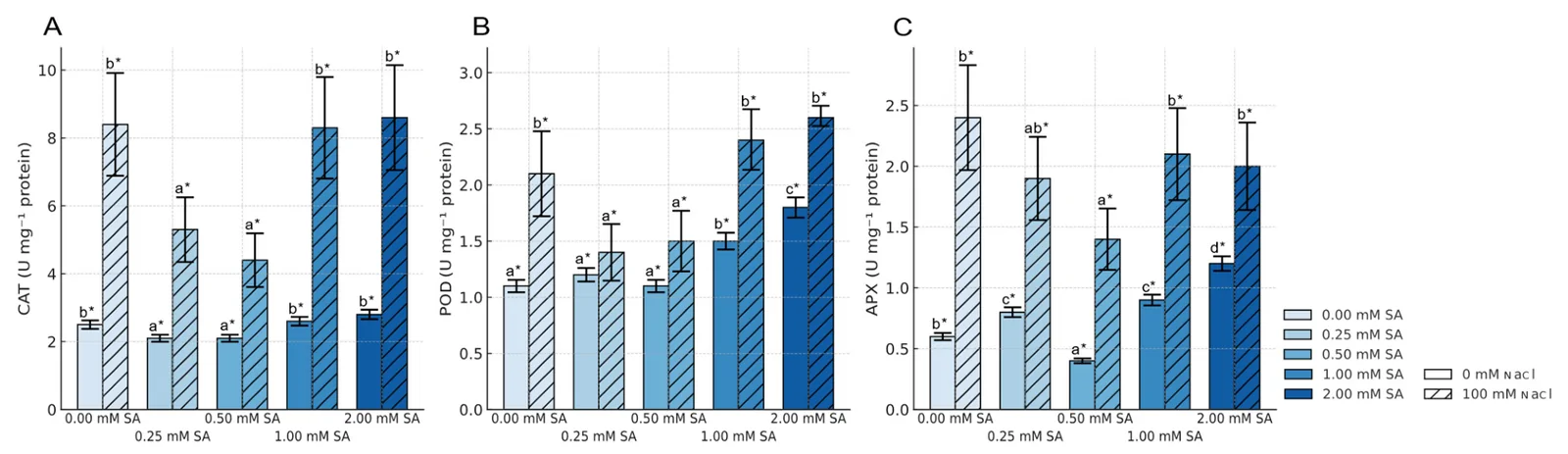
Enzymatic activity of catalase (CAT) (**A**), peroxidase (POD) (**B**), and ascorbate peroxidase (APX) (**C**) measured in *Calendula officinalis* leaves grown under two NaCl levels (0 mM and 100 mM) and five SA concentrations (0.00, 0.25, 0.50, 1.00, or 2.00 mM). Bars represent mean ± SE (*n* = 10). Different lowercase letters indicate significant differences among SA concentrations within the same NaCl level, whereas asterisks indicate significant differences between NaCl levels within the same SA concentration (*p* < 0.05).

Salinity significantly increased CAT activity (F_1,90_ = 22.54, *p* < 0.001; Figure 5). These effects changed with the application of SA although depending on its dose (F_4,90_ = 5.21, *p* < 0.001). Under non-saline conditions, CAT activity remained relatively stable and as even reduced at 0.25 and 0.50 mM SA, increasing again at highest SA concentrations (1.00 and 2.00 mM) (Figure 5). Under saline conditions, 0.25 and 0.50 mM SA significantly reduced CAT activity relative to the saline treatment without SA, whereas at 1.00 and 2.00 mM SA, CAT activity increased again to levels statistically comparable to the saline treatment without SA (Figure 5).

Salinity also affected the activity of POD (F_1,90_ = 11.02, *p* < 0.001) as well as the application of SA (F_4,90_ = 4.39, *p* < 0.001) (Figure 5). Under non-saline conditions, activity remained unchanged from 0 to 0.50 mM SA increasing only at highest SA concentrations (Figure 5). Under salinity, 0.25 and 0.50 mM SA reduced POD activity while 1.00 and 2.00 mM SA increased it again, reaching the levels observed in the saline treatment without SA (Figure 5).

A similar pattern was detected in APX activity with salinity strongly increasing its activity (F_1,90_ = 15.11, *p* < 0.001) (Figure 5). Under non-saline conditions, APX activity increased at 0.25 mM SA, was significantly reduced at 0.50 mM SA and increased again at 1.00 and 2.00 mM SA. Under saline conditions, APX activity declined following application of 0.25 mM SA and reached its lowest value at 0.50 mM SA, before increasing again at 1.00 and 2.00 mM SA to levels statistically comparable to the saline treatment without SA (F_4,90_ = 22.19, *p* < 0.001; Figure 5).

Overall, the significant NaCl × SA interactions reflected a concentration-dependent modulation of antioxidant enzyme activities (CAT: F_4,90_ = 23.42, *p* < 0.001; POD: F_4,90_ = 22.76, *p* < 0.001; APX: F_4,90_ = 25.08, *p* < 0.001). Notably, under salinity, 0.50 mM SA reduced CAT, POD, and APX activities by approximately 48%, 29%, and 42%, respectively, relative to the saline treatment without SA.

### 3.6. Abscisic Acid (ABA) Content

**Figure 6:**
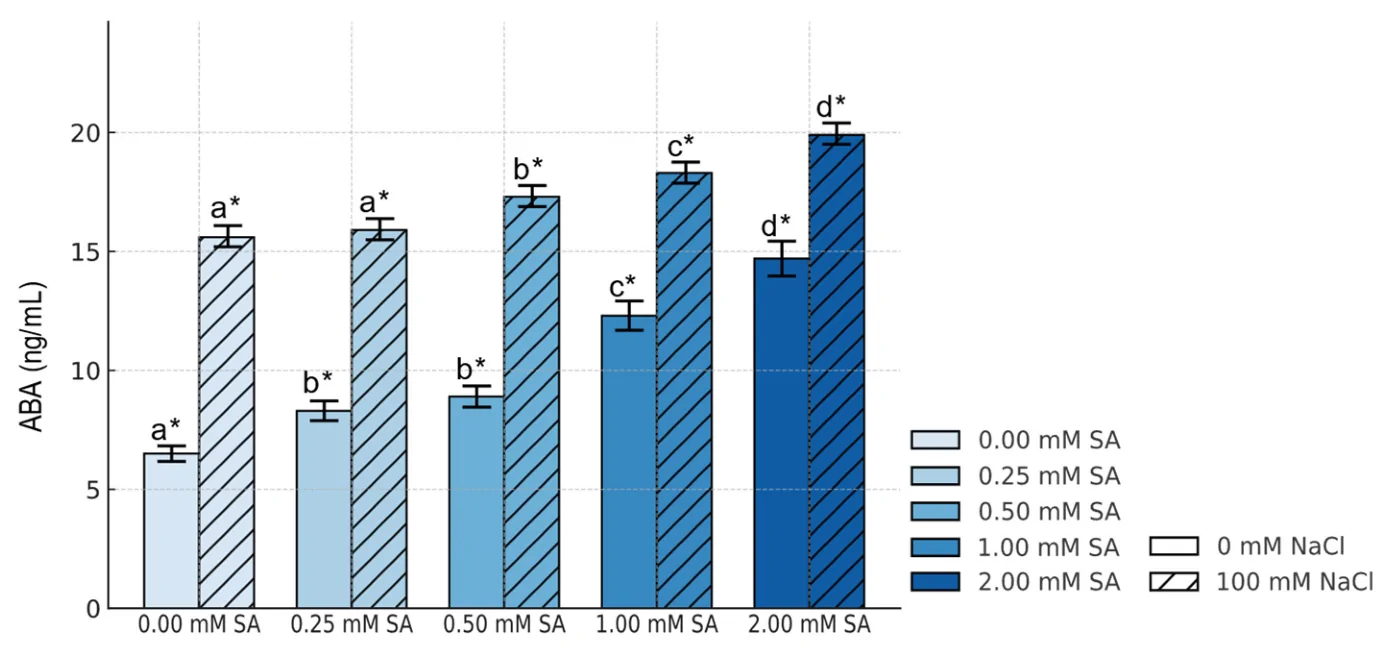
Abscisic acid (ABA) content measured in leaves of *Calendula officinalis* grown under two NaCl levels (0 mM and 100 mM) and five SA concentrations (0.00, 0.25, 0.50, 1.00, or 2.00 mM). Bars represent mean ± SE (*n* = 10). Different lowercase letters indicate significant differences among SA concentrations within the same NaCl level, whereas asterisks indicate significant differences between NaCl levels within the same SA concentration (*p* < 0.05).

Salinity significantly increased ABA content (F_1,90_ = 62.27, *p* < 0.001; Figure 6). SA concentration also significantly affected ABA content (F_4,90_ = 21.82, *p* < 0.001). Under non-saline conditions, ABA content increased progressively with increasing SA concentration, with the highest values observed at 2.00 mM SA (Figure 6). A similar concentration-dependent increase was observed under salinity, although ABA levels were consistently higher than those of the corresponding non-saline treatments (Figure 6). A significant NaCl × SA interaction was detected on ABA contents (F_4,90_ = 28.14, *p* < 0.001) suggesting that the strong salinity-induced increase in ABA was also affected by SA concentration.

## 4. Discussion

As environmental change reshapes agricultural systems worldwide, maintaining reproductive performance under salinity has become as important as preserving vegetative growth. In this study, *C. officinalis* plants exposed to 100 mM NaCl exhibited pronounced stress symptoms, including significant reductions in plant height, leaf and flower number, flower diameter, and pigment content, together with marked decreases in fresh and dry biomass. These phenotypic impairments were accompanied by a decline in relative water content (RWC), together with increased proline accumulation, enhanced lipid peroxidation (MDA), and strong upregulation of antioxidant enzyme activities (CAT, POD, and APX). Collectively, these responses are consistent with the well-established effects of salinity stress, which involve osmotic, ionic, and oxidative components [46,47,48,49,50], but also show the potential of SA as a promising strategy to mitigate these effects. More importantly, our findings highlight that assessing vegetative responses alone is insufficient to understand plant performance under salinity. By placing flowering at the centre of the analysis and integrating it with physiological, biochemical, and hormonal responses, this study provides a comprehensive framework of the effects of salinity on plants.

### 4.1. SA-Mediated Alleviation of Salinity Effects

Salinity stress is known to impair plant performance at multiple levels, from vegetative growth to reproductive development [51], and *C. officinalis* was no exception. Plants subjected to 100 mM NaCl showed marked reductions in height, leaf and flower number, flower diameter, and pigment content, together with significant decreases in both fresh and dry biomass. These morphological impairments are typical outcomes of osmotic stress, ionic toxicity, and oxidative damage [52,53]. In particular, the sharp decline in flower number and size highlights the sensitivity of reproductive structures to salinity. Under salinity, untreated plants showed steep reductions in both fresh and dry weight, as well as RWC, reflecting physiological performance and growth [17]. Similar findings in other ornamentals, where salinity curtailed floral initiation and longevity [12,54,55], reinforce the notion that reproductive traits are often the first to decline under stress, making them critical targets for mitigation strategies.

The application of exogenous SA alleviated some of these detrimental effects, but the extent of protection depended on the concentration used. At low doses (0.25–0.50 mM), SA-treated plants maintained greater height, leaf number, and floral output compared with salt-stressed controls, and even developed traits that approached those in non-saline conditions or even surpassed them. These improvements were accompanied by significant increases in biomass production, indicating that the beneficial effects of moderate SA concentrations extended beyond reproductive traits to whole-plant growth [56]. By contrast, supra-optimal SA concentrations (1.00–2.00 mM) failed to confer similar benefits, and in some cases further reduced morphological and reproductive performance as in other plants [57]. This biphasic response is typical of plant signaling molecules, which act as regulators rather than simple growth stimulants, and it underscores the importance of precise dose optimization [58].

An interesting feature of our results was the relationship between water status and osmolyte accumulation. Salinity markedly reduced RWC, indicating substantial osmotic stress, while simultaneously promoting strong proline accumulation, a common response to water deficit [41,59]. Application of 0.50 mM SA substantially improved RWC and was accompanied by lower proline accumulation than in untreated salt-stressed plants. This pattern suggests that SA reduced the severity of osmotic stress and, consequently, the requirement for extensive osmolyte accumulation. Given the metabolic cost associated with proline synthesis, the combination of higher RWC and lower proline levels may indicate a more efficient maintenance of plant water status under SA.

At the biochemical level, the oxidative stress profile further illustrates the mitigating role of SA. Salinity induced a sharp rise in MDA, confirming enhanced lipid peroxidation and membrane destabilization, and triggered strong upregulation of antioxidant enzyme activities, including CAT, POD, and APX. While activation of these enzymes reflects an attempt to detoxify ROS [36,60], their overinduction suggests that plants were under oxidative pressure and allocating substantial resources to damage control rather than to growth. SA application markedly altered this response. At moderate doses, MDA levels declined and antioxidant enzyme activities (CAT, POD, and APX) were reduced compared with salinity alone. Importantly, the lower MDA content provides direct evidence of reduced lipid peroxidation, whereas the accompanying changes in enzyme activities indicate that SA modified the antioxidant response to salinity [59]. At higher concentrations, however, SA lost its protective effect, as reported previously in chickpea (*Cicer arietinum*) plants [61]. This decline in efficacy may be partly explained by adverse effects of excessive SA on photosynthetic performance, as stomatal conductance (gs) has been shown to decrease progressively with increasing SA concentrations as reported in other studies [62]. Consistent with this interpretation, MDA levels increased again at the highest SA concentrations in our study, while antioxidant enzyme activity patterns became similar to those observed under NaCl alone. Excessive SA has also been reported to promote peroxisomal H_2_O_2_ accumulation [63], which could contribute to the growth inhibition observed at 1.00–2.00 mM SA. This response has been linked to SA-mediated modulation of key H_2_O_2_-scavenging enzymes, including catalase (CAT) and cytosolic ascorbate peroxidase [64]. Although our results do not support such an effect for CAT, we cannot exclude the involvement of other antioxidant enzymes or hormone-mediated signalling pathways. Overall, our results indicate that the beneficial effects of SA are strongly dose-dependent, supporting its role in coordinating antioxidant responses and stress signalling when applied at appropriate concentrations.

Notably, beyond its beneficial effects on vegetative performance, SA exerted a clear protective effect on flowering, particularly by preserving flower number and diameter under saline conditions. While numerous studies have demonstrated that SA improves vegetative growth, comparatively few have examined its capacity to maintain reproductive performance under salinity. In *C. officinalis*, [65] reported that foliar SA application (1.00, 2.00 mM) alleviated salinity-induced reductions (100 and 200 mM) of several traits including flower production, especially with 1.00 mM SA. Likewise, the combined foliar application of SA and fulvic acid, particularly at 200 mg L^−1^ SA and 3 g L^−1^ fulvic acid, significantly increased flower number and flower longevity in *C. officinalis* grown under saline conditions [66]. Together, these studies support the beneficial effects of SA on flowering under saline conditions. Our findings build on this evidence by showing that these reproductive responses occur in parallel with coordinated physiological, biochemical, and hormonal adjustments, providing a broader framework for understanding the conditions under which SA mitigates salinity-induced reductions in flowering traits.

### 4.2. ABA Responses Associated with SA Application Under Salinity

Hormonal regulation is at the heart of plant adaptation to abiotic stress, and our results suggest the involvement of ABA-associated responses in the effects of SA under salinity *C. officinalis*. Both salinity and SA independently increased leaf ABA content, but the highest levels were observed when the two were combined, suggesting that exogenous SA is associated with enhanced ABA accumulation under salinity. This is a particularly significant finding, as ABA is widely regarded as a stress hormone coordinating plant responses to stress mainly through stomatal closure, osmotic adjustment, and induction of stress-related genes [67,68]. ABA also induces the expression of late embryogenesis abundant (LEA) proteins, dehydrins, and aquaporins, which help preserve cell turgor and protect proteins and membranes during osmotic stress [15]. Previous studies have also reported that SA can influence the transcription of NCED genes, which encode 9-cis-epoxycarotenoid dioxygenases involved in ABA biosynthesis [69,70]. SA has been associated with enhanced abscisic aldehyde oxidase activity, which catalyzes the final step of ABA production [71]. SA could also, hypothetically, interact with ABA signaling components, including PYR/PYL/RCAR receptors and SnRK2 kinases, thereby amplifying ABA responses [72], although these mechanisms were not assessed in the present study and should therefore be interpreted with caution.

Here, the higher ABA accumulation observed under combined SA and salinity treatment occurred alongside changes in water status, pigment content, and oxidative stress-related responses. Interestingly, SA-treated plants showed lower proline accumulation despite higher ABA levels, indicating that ABA accumulation and proline responses were not quantitatively coupled under these conditions. Overall, our findings suggest that the beneficial effects of SA are associated with enhanced ABA accumulation together with improved water status and redox homeostasis. These observations are consistent with the involvement of ABA-associated responses, although the molecular mechanisms underlying SA–ABA interactions remain to be elucidated.

### 4.3. Implications, Gaps, and Ecological Considerations

Beyond mechanistic insights, the results of this study carry several important implications for both fundamental plant science and applied horticulture, supporting the idea that novelty in plant stress biology increasingly resides in understanding how multiple physiological and signaling pathways are coordinated, rather than in the identification of individual responses in isolation. From a practical perspective, the use of SA represents a low-cost and easily applicable compound that could be integrated into ornamental crop management as a biostimulant, enhancing plant resilience and preserving visual quality under saline conditions [73]. Unlike most previous work that were primarily focused on vegetative traits, our findings demonstrate that SA mitigated the effects of salinity on flowering, particularly flower number and size, which are the main determinants of ornamental value. This represents a novel contribution to the field, as the preservation of floral quality under stress has rarely been documented, but see [12], which is particularly relevant given the growing recognition that reproductive structures represent critical bottlenecks in plant responses to environmental stress [1].

Despite these advances, several important knowledge gaps remain to be further addressed. One of the immediate challenges concerns the narrow protective window of SA, since as reported here, moderate concentrations improved plant performance while higher doses caused phytotoxic effects. This emphasizes the need for further research on dose optimization, timing, and frequency of application to maximize benefits while avoiding stress exacerbation [56,74]. For instance, additional studies evaluating ion homeostasis, root responses, photosynthetic gas exchange, direct ROS quantification, and endogenous SA dynamics will contribute to a more comprehensive understanding of the underlying mechanisms. Another critical step is the validation of these findings under field conditions, where plants are exposed to fluctuating salinity levels and the simultaneous action of multiple stresses [60]. At the mechanistic level, it is unclear whether floral organs exhibit unique sensitivities to SA and ABA compared with vegetative tissues, and whether the beneficial effects observed here for flower number and size extend to reproductive fitness traits such as pollen viability and seed production [75]. Finally, varietal variability within *Calendula* and the general applicability of SA across other ornamental species remain open questions [76], as does the potential role of SA priming in conferring cross-tolerance to combined stresses [77]. Addressing these questions will be critical to fully harness the potential of SA in both fundamental research and applied horticultural practice.

In addition to plant-level effects, the ecological footprint of SA use warrants careful consideration. Foliar-applied SA inevitably reaches the soil through runoff, leaching, or incorporation of plant residues, where it can interact with microbial communities and nutrient cycles. As a phenolic compound, SA may act as a signaling molecule in the rhizosphere, potentially influencing the composition of microbial assemblages by favoring some taxa while suppressing others [78]. This raises further questions that remain largely unexplored: How long does SA persist in soils following repeated applications, and at what concentrations? Does it selectively enhance antagonistic microorganisms that suppress pathogens, or could it inadvertently inhibit beneficial symbionts such as mycorrhizal fungi and nitrogen-fixing bacteria? Could repeated inputs alter nutrient cycling processes, such as nitrogen mineralization or phosphorus availability, thereby impacting long-term soil health? Addressing these uncertainties will require integrative approaches combining plant physiology, molecular biology, and soil ecology. Only through such multidimensional research can we evaluate the sustainability of SA as a biostimulant and ensure that its use enhances plant resilience without compromising soil function and ecosystem services.

## 5. Conclusions

This study provides physiological, biochemical, and hormonal evidence associated with the beneficial effects of SA on flowering under salinity. SA mitigated salinity-induced growth and flowering inhibition and maintained higher RWC and higher chlorophyll and carotenoid contents under salinity. These effects were accompanied by reduced lipid peroxidation together with a modulation of osmolyte accumulation and antioxidant enzyme activities. Importantly, SA application also modulated ABA accumulation, and these changes were associated with the responses observed under salinity. From an applied perspective, these findings provide a basis for integrating SA treatments into ornamental crop management strategies, with potential for maintaining flower production under saline irrigation regimes. Further research should examine dose–response thresholds and the long-term impacts of repeated SA applications to optimize their agronomic use and evaluate the sustainability of SA as a biostimulant.

## Data Availability

The datasets generated during and/or analyzed during the current study are available within the paper.
